# Assessment of the readiness of restorations manufactured by CAD/CAM in terms of marginal fit (Part I)

**DOI:** 10.7717/peerj.13280

**Published:** 2022-05-02

**Authors:** Radek Mounajjed, Thomas Taylor, Omar Hamadah, Iva Voborná, Marwan Al-akkad

**Affiliations:** 1 DCM Clinic, Hradec Kralove, Czech Republic; 2 Institute of Dentistry and Oral Sciences, Faculty of Medicine and Dentistry, Palacký University Olomouc, Olomouc, Czech Republic; 3 Department of Reconstructive Sciences, UConn School of Dental Medicine, Farmington, Connecticut, United States of America; 4 Department of Oral Medicine, Faculty of Dental Medicine, Damascus University, Damascus, Syria

**Keywords:** CAD/CAM, Marginal discrepancy, Digitalization, Digital workflow, Metal coping, Master cast, Marginal gap, Metal substructures, Digital impression, Marginal adaptation

## Abstract

**Background:**

The master cast is the gold standard for the control and eventual adjustment of restorations produced by conventional procedures. Some digital workflow bypasses the master cast and relies completely on the precision of the CAD/CAM restoration.

**Aim:**

To examine the reproducibility of the margins of CAD/CAM restorations generated from a single digital scan. Also, to check the readiness of these restorations for delivery directly after fabrication without adjustment on a master cast and thereby eliminate the need for the master cast.

**Methods:**

A total of 18 metal substructures made from cobalt chrome alloy were fabricated utilizing a single STL file. The circumference was divided into eight zones. The vertical marginal discrepancy (VMD) was measured at each zone of each metal substructure, with optical microscopy at ×200 magnification.

**Results:**

Measurements of vertical marginal discrepancy were in a range of (−94: 300) with a mean of 62 ± 60 μm. A one-way ANOVA test revealed that the mean VMD is significantly different among the 18 substructures (F17, 1,134 = 63.948, *p* < 0.001).

**Conclusion:**

Although all the received substructures were fabricated from the same scan file, they were not identical and varied widely, and they were going outside the acceptable range in some zones. Within the limitations of this study, the marginal fit can be improved by extraoral adjustments on the master cast. Thus, skipping the master cast deprives the dentist of delivering a restoration of higher quality.

## Introduction

Computer-aided design/Computer-aided manufacturing (CAD/CAM) in dentistry is defined as using computer technology to design and produce different types of dental restorations (Grant et al., 2016). This technology is rapidly increasing the use of digital workflow for dental prosthesis fabrication (Lo Russo et al., 2019). Digital scans offer not only a fast, efficient process but also the ability to store captured information indefinitely and to transfer them easily between the clinic and the laboratory (Yuzbasioglu et al., 2014). To initiate the digital workflow for a CAD/CAM system, either direct or indirect digitalization techniques can be used (Güth et al., 2017). In the direct digitalization technique, the prepared tooth is scanned in the oral cavity by an intraoral optical scanner (Beuer, Schweiger & Edelhoff, 2008), while in the indirect digitalization technique a conventional silicone impression is made first then the impression or cast is scanned with an extraoral scanner (Vecsei et al., 2017). Then, the acquired data are used for the designing (CAD) and manufacturing (CAM) of the prosthetic restoration (Güth et al., 2016).

The importance of the master cast is not only limited to obtaining a digital scan during the CAD phase but also to control the margins after the CAM phase and to adjust them to improve their quality. Despite the ability to do the adjustments directly in the patient’s mouth, the extraoral adjustment on a master cast gives superior results. In addition to being more convenient for the patient and less demanding for the dentist, it gives the dental technician a chance to evaluate their work and modify it accordingly.

### Marginal fit

Marginal fit is a key factor in the success of restorations (Mounajjed, Layton & Azar, 2016; Mounajjed et al., 2018). The best method to measure marginal gaps remains a controversial topic (Riccitiello et al., 2018). Moreover, the terminology describing “fit” along with the techniques used for measuring fit vary considerably in the literature.

**Figure 1:**
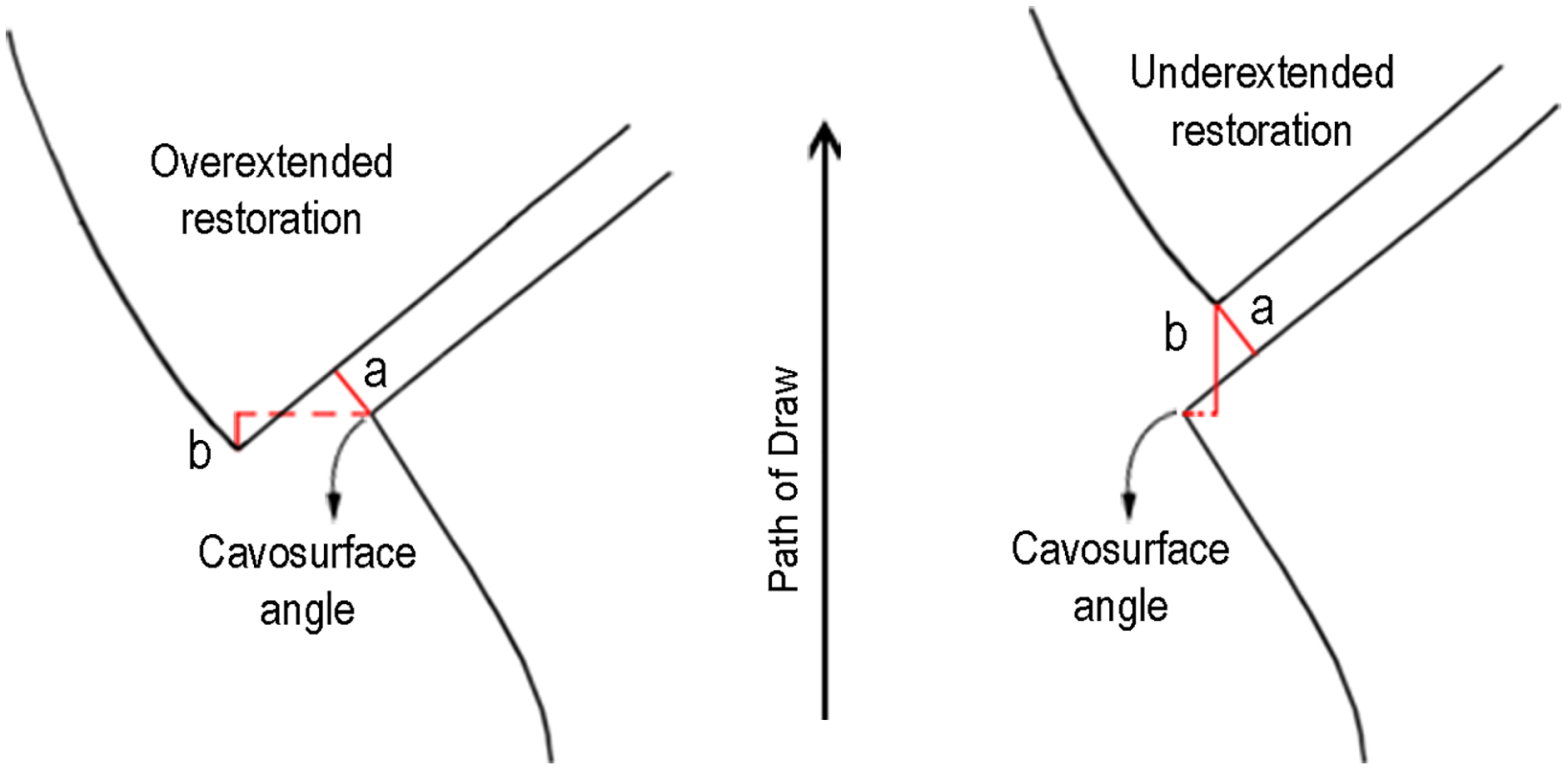
Marginal misfit. (A) Marginal gap. (B) Vertical marginal discrepancy. (Adapted from Holmes et al. (1989), © 1989 The C.V. Mosby Company. Published by Elsevier Inc.).

From a practical aspect, a proper description of the fit of a restoration can be achieved by describing the different types of misfits which exist in that restoration and by providing measurements of each type of these misfits. These measurements are provided in micrometers. It is critical for these measurements to be done systematically since there are many different locations between a tooth and a restoration where the measurements can be made (Holmes et al., 1989). The vertical marginal discrepancy (VMD) is defined as the vertical marginal misfit measured parallel to the path of the draw of the casting (Holmes et al., 1989) (Fig. 1). The value of the VMD is directly related to both the marginal gap and to the margins of the metal substructure being underextended or overextended.

In the case of under extension, the VMD is always greater or equal to the marginal gap and the substructure margin is always coronal to the cavo-surface angle of the tooth. In the case of overextension, the vertical marginal discrepancy is always accompanied by horizontal marginal discrepancy and the cavo-surface angle of the tooth is always coronal to the substructure margin (Fig. 1).

The marginal gap should, in theory, be small enough to prevent the ingress of saliva and/or lactic acid, which is the byproduct of bacterial metabolism (Azar et al., 2018). A mean marginal gap of less than 100 μm has been considered clinically acceptable for CAD/CAM restorations (Svanborg et al., 2014; Freire et al., 2021).

An overextended margin promotes accumulation of plaque, increases the number of specific periodontal pathogens in the plaque, and hinders flossing in the interproximal embrasure potentially leading to periodontal disease (Brunsvold & Lane, 1990; Millar & Blake, 2019). The response of periodontal tissues appears to relate primarily to overextension rather than to an increased marginal gap (Boeckler, Stadler & Setz, 2005). When overextended margins are removed, the control of plaque can be performed more effectively (Yasar, Yesilova & Akgünlü, 2010).

Large marginal gaps can result from the internal misfit of the restoration substructure (Fig. 1). In this case, internal adjustment of the substructure with a pressure paste could reduce the marginal gap. On the other hand, overextended margins are adjusted by trimming these overextended margins to the level of the finish line by using a rotary instrument. VMD is a single value that indicates the demand for an adjustment in both cases, which can only be done on a master cast acquired from the indirect digitalization technique.

This study was conducted to answer the following question: Are restorations manufactured by CAD/CAM ready to be used directly after fabrication without adjustment on the master cast and consequently eliminating the need for the master cast?

This question emerged from the daily clinical observation where clinicians in the DCM clinic noticed that such minimal adjustments are necessary in most cases.

**Figure 2:**
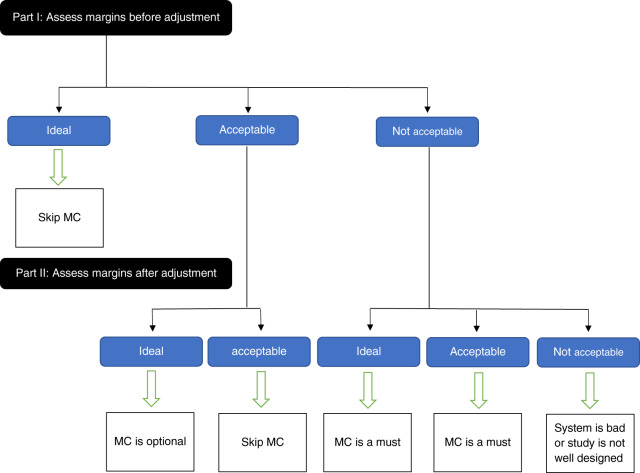
Road map to answer the question: *are we ready to skip the master cast?* To answer this large question, a roadmap that goes through consequent sub-questions was created. MC: Master Cast.

To answer this large question, a roadmap that goes through consequent sub-questions was created. The first sub-question, how fit are the margins of the metal substructure manufactured by indirectly digitalized CAD/CAM? If the answer is perfectly fit, then the master cast can be definitely skipped. Otherwise, we need to investigate a second sub-question in a subsequent study, are the margins of the metal substructure manufactured by CAD/CAM after adjusting on the master cast fitting better? Based on this answer we can determine the importance of the master cast (Fig. 2).

Furthermore, clinicians in the DCM clinic claim, based on their observation, that the quality of the restorations might be related to the day of the week in which the scan was made. They claimed that on the first working day of the week, restorations have high quality then it decreases gradually to be the worst on the last working day of the week. Determining that a single STL file can identically replicate repeated restorations is a key factor in this framework.

The null hypothesis was that the margins of the metal substructure manufactured by CAD/CAM are neither overextended nor associated with a marginal gap equal to or greater than 100 μm. The second null hypothesis was that there is no correlation between the quality of the metal substructure manufactured by CAD/CAM and the day of the week in which the milling process was made.

## Materials and Methods

### Preparations before microscopic examination

An artificial acrylic mandibular right first permanent molar (tooth #46) was prepared to receive a porcelain fused to metal (PFM) crown on a mandibular dental model with elastic replacement anatomical gingiva (Model AN-4 WUK; Frasaco, Tettnang, Germany). The justification for choosing the metal substructure of a PFM crown to study the marginal discrepancy is the elimination of factors affecting the marginal fit that are not related to the CAD/CAM process such as interproximal contact with the adjacent teeth.

**Figure 3:**
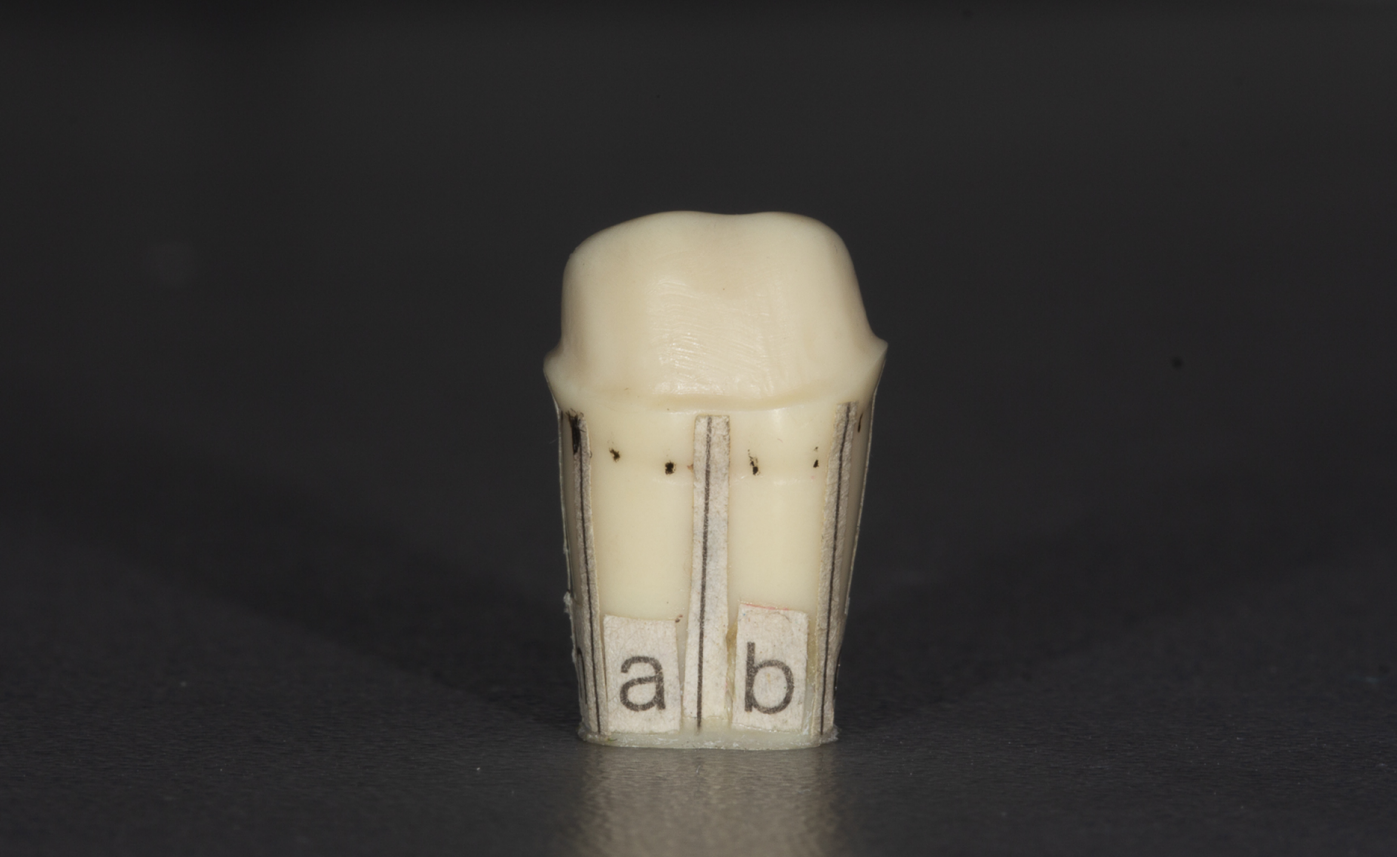
Prepared tooth without any metal substructure.

**Figure 4:**
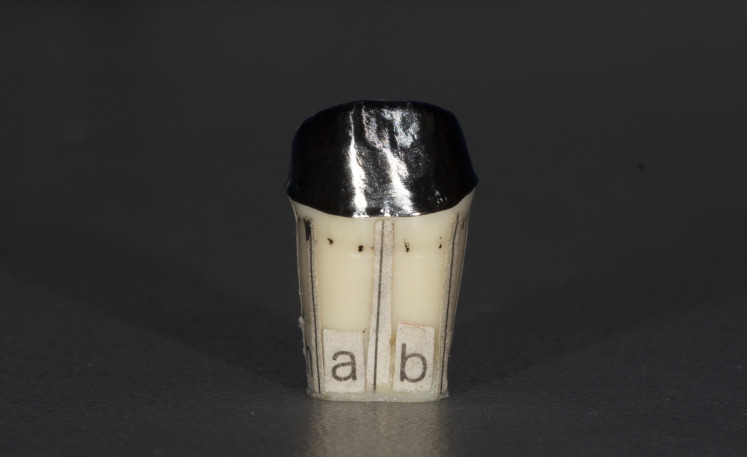
Prepared tooth with one metal substructure.

The line of the draw was identical to the long access of the tooth. The finish line was chamfered 0.5 mm. The circumference of the external edge of the finish line was 39.6 mm. The model was considered to represent the master cast. The justification for preparing the cast itself rather than preparing a tooth in a patient’s mouth was to eliminate potential human error from the physical impression process such as dimensional changes of impression and casting materials. A scan to the prepared tooth within the dental model was done by using an extraoral dental scanner (Straumann® CARES® 7Series). The same scan was sent to the dental laboratory (Straumann® CARES® CAD/CAM; Centralized Milling Center, Germany) 18 separate times using different patient names each time. The laboratory was not informed about the study. However, upon requesting the laboratory representative about the process, it was clearly explained that no 3D model of the scanned tooth is printed to make any adjustments before delivery of the restorations. To ensure randomization, a virtual dice (Dice version 8.1 from SeableApps) was rolled twice at the beginning of each week to choose the days on which the scan was sent to the laboratory (Table 1). The tooth was divided into eight zones: A and B zones for the buccal surface, C zone for mesio-buccal surface, D zone for mesio-lingual surface, E and F zones for lingual surface, G zone for disto-lingual surface, and H zone for disto-buccal surface (Fig. 3). Cobalt-chrome alloy (Coron®) was used to produce the metal substructures (Fig. 4). The tooth was mounted on a fixed glass plate on a high-precision manual rotation mount (PRM1/M; Thorlabs, Inc., Newton, NJ, USA) that can rotate 360° to be studied under the microscope from different angles.

**Table 1:** Randomization of the substructures.

Dices		Week no in 2019	M	Tu	W	Th
⚂	⚅	12			✓	
⚀	⚁	13	✓	✓		
⚀	⚃	14	✓			✓
⚅	⚅	15				
⚀	⚂	16	✓		✓	
⚄	⚃	17				✓
⚁	⚃	18		✓		✓
⚄	⚅	19				
⚁	⚄	20		✓		
⚀	⚃	21	✓			✓
⚄	⚅	22				
⚃	⚅	23				✓
⚀	⚂	24	✓		✓	
⚄	⚄	25				
⚂	⚄	26			✓	
⚀	⚅	27	✓			
Total			6	3	4	5

After receiving the 18 fabricated metal substructures ready for the try-in stage (*n* = 18), they were studied on the mounted tooth at 200× magnification under digital optical microscopy (VHX-5000; Keyence, Osaka, Japan) at the Department of Experimental Physics in the Faculty of Science in Palacký University Olomouc. The microscope is equipped with an acquisition camera with a resolution of 1,600 × 1,200 pixels and has a special image analyzing software supporting it.

### Measurement of VMD

The microscope captured two distinctive photomicrographs from each zone for each substructure resulting in 288 photomicrographs. The revolving base was rotated 22.5° each time to cover the full 360°.

VMD is defined as the distance from a determined point on the finish line to the closest point on the margin of the substructure. VMD was measured from four different points in each photomicrograph resulting in 1,152 measurements. The justification for this large number of measurements comes from a study that recommended a minimum of 50 measurements per crown (Groten et al., 2000).

To distinguish between VMD in the case of overextended margins and in case of underextended margins, the term forward vertical marginal discrepancy (Forward VMD) is used in case of overextension while the term backward vertical marginal discrepancy (Backward VMD) is used in case of under-extension. Forward VMD was recorded as a negative value, whereas Backward VMD was recorded as a positive value.

All the measurements were done by the same examiner who took all the photomicrographs.

Since each zone was measured at eight different points, the level of VMD in each zone was represented by a number in a scale from 0 to 8, where 0 means that no measurement in that zone was outside the acceptable range of 0 to 100 μm, while one means that one measurement was outside the acceptable range and so on. The levels of Forward VMD and Backward VMD beyond 100 μm were calculated similarly. In this regard, the level of VMD is the sum of the level of Forward VMD and the level of Backward VMD beyond 100 μm.

**Figure 5:**
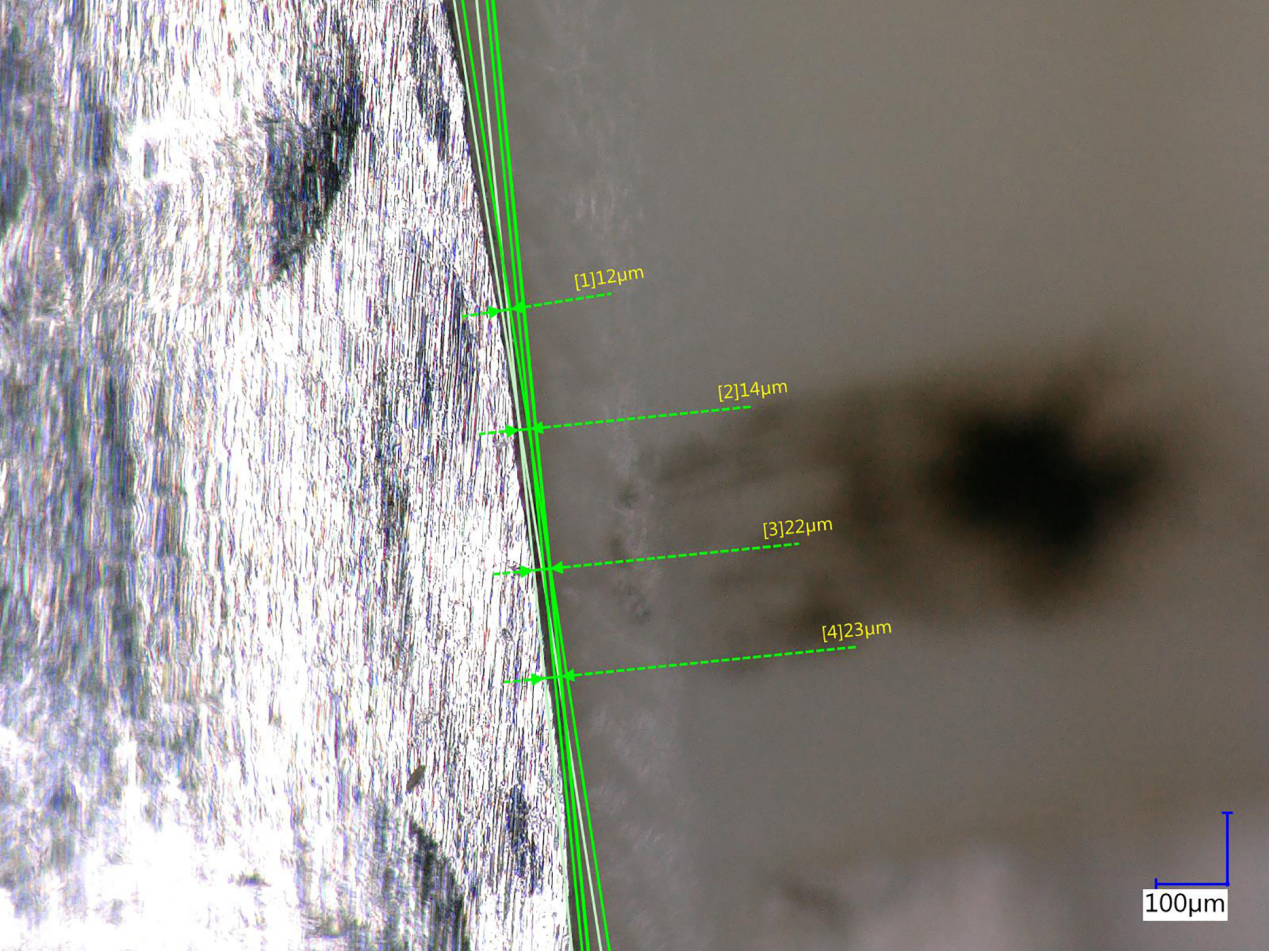
Photomicrograph of overextended margin.

**Figure 6:**
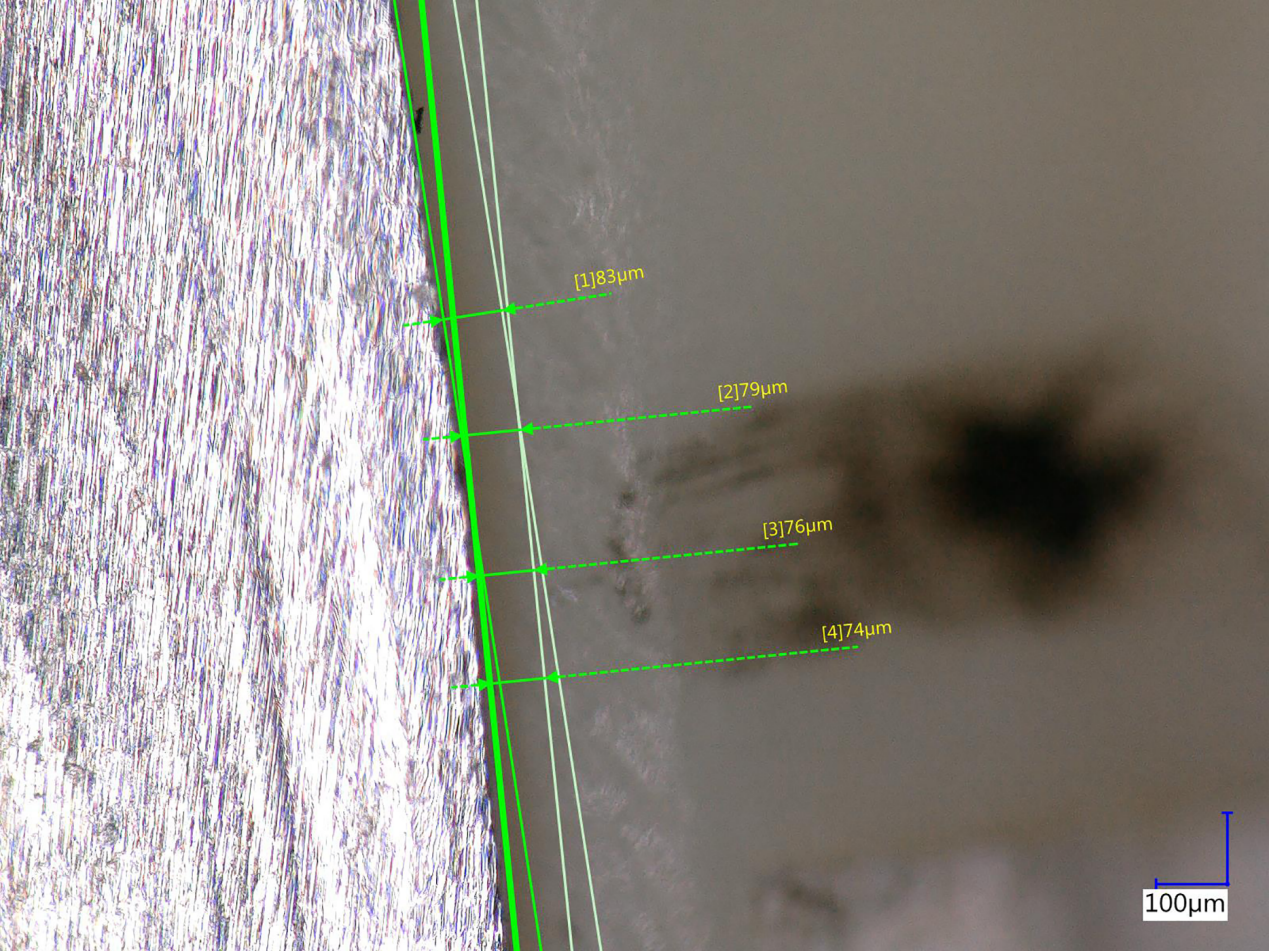
Photomicrograph of underextended margin.

Figure 5 is a representative photomicrograph for Forward VMD. Figure 6 is a representative photomicrograph for Backward VMD.

After receiving and measuring all the 18 substructures, the laboratory was asked to provide the exact dates of milling.

### Statistical analysis

Several spreadsheets (Microsoft Excel, MS Office 2019; Microsoft, Redmond, WA, USA) were used to record the measurements, to document scanning and milling dates of the substructures along with their serial numbers, and to calculate the levels of VMD, Forward VMD, and Backward VMD beyond 100 μm.

All statistical tests were carried out by using the Statistical Package for the Social Sciences – SPSS version 27.0 (SPSS Inc., Chicago, IL, USA, 2020). Primarily, descriptive statistics were used to test the normality of data, and the measurements of each zone were represented by frequencies (N), percentages (%), mean ± standard deviation (SD), and range. Consequently, inferential statistics were used to evaluate the correlation between the level of Forward VMD and the level of Backward VMD beyond 100 μm in all the zones, and between the zones themselves in terms of level of VMD. Spearman rank-order correlation coefficient test (Spearman’s correlation) was used with a significance level of <0.05; and the levels of correlation were depicted as negligible correlation (0–0.1); weak correlation (0.10–0.39); moderate correlation (0.40–0.69); strong correlation (0.7–0.89); very strong correlation (0.90–1).

## Results

**Figure 7:**
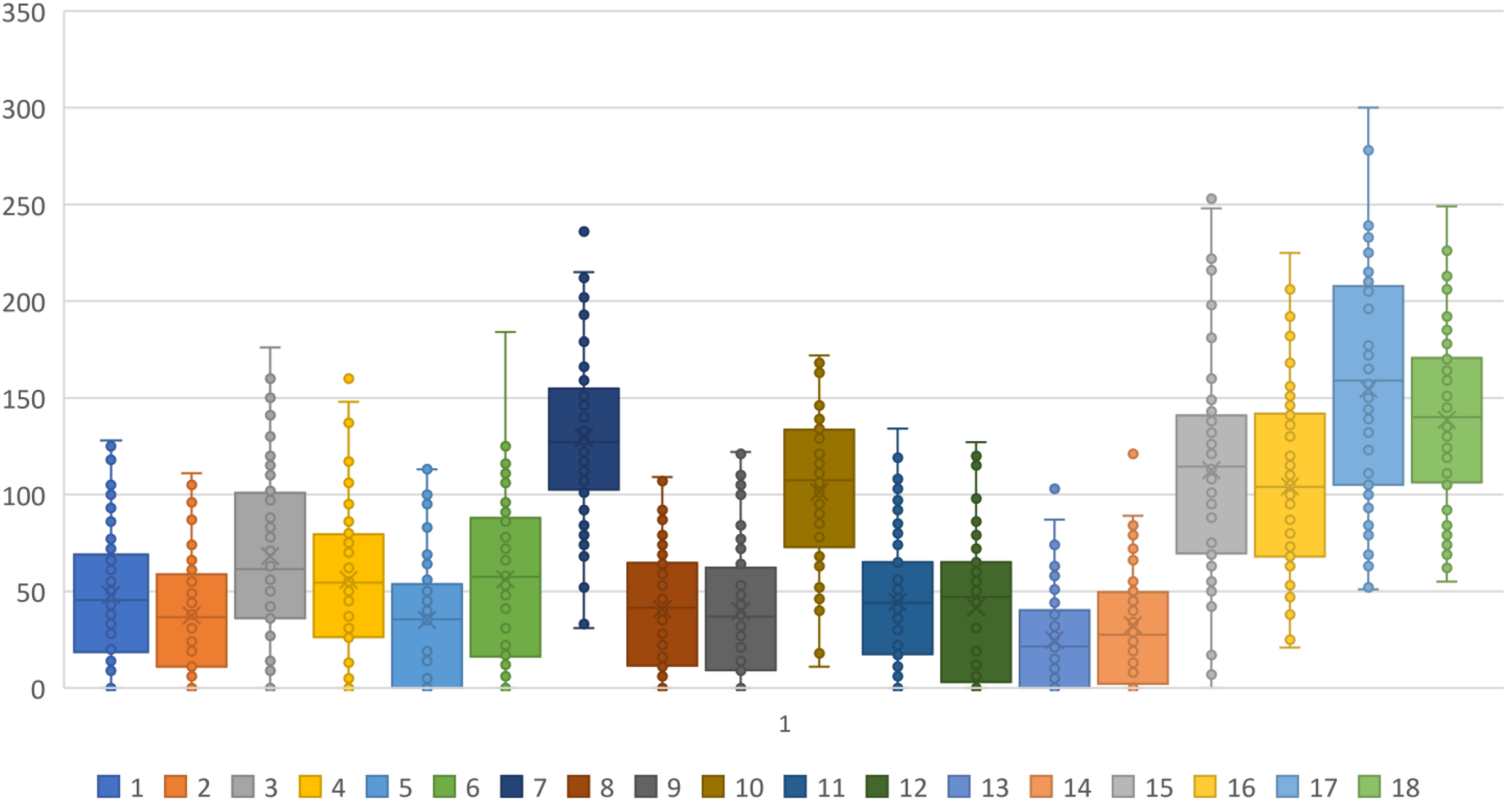
Boxplot of the backward VMD of the 18 substructures.

Mean measurements and standard deviation of the metal substructures were stratified by zones (A–H) in Tables 2–4. A one-way ANOVA test reveals that the mean VMD is significantly different among the 18 substructures (F17, 1,134 = 63.948, *p* < 0.001). Backward VMD was not greater than 100 μm as per Single Sample *t* Test. A boxplot of the Backward VMD of the 18 substructures is illustrated in Fig. 7.

**Table 2:** Mean measurements of the metal substrcutrues stratified by zones (A–C).

	Zone A	Zone B	Zone C
ID	μ ± SD (min : max)	μ ± SD (min : max)	μ ± SD (min : max)
1	47.36 ± 34.8 (2 : 78)	89.38 ± 24.02 (66:128)	55.38 ± 32.26 (15 : 125)
2	9.25 ± 36.7 (−53 : 47)	73.25 ± 18.33 (55 : 100)	49.38 ± 30.91 (2 : 105)
3	54.25 ± 56.01 (−20 : 110)	134.5 ± 26.55 (106 : 176)	88.25 ± 32.78 (54 : 160)
4	35.88 ± 42.93 (−26 : 83)	104 ± 28.15 (76 : 148)	83.88 ± 33.46 (56 : 160)
5	7.75 ± 46.44 (−63 : 56)	70.75 ± 25.93 (44 : 113)	45.13 ± 21.58 (19 : 87)
6	41.38 ± 46.16 (−30 : 88)	97.88 ± 14.52 (86 : 125)	75.13 ± 54.04 (20 : 184)
7	113.13 ± 55.36 (31 : 170)	188.63 ± 38.25 (152 : 238)	147.75 ± 31.26 (117 : 212)
8	0.75 ± 48.37 (−72 : 53)	66.13 ± 25.85 (40 : 107)	49.25 ± 28.78 (18 : 108)
9	11.25 ± 52.07 (−62 : 65)	70.88 ± 25.91 (43 : 110)	66.5 ± 28.75 (31 : 122)
10	81.63 ± 45.61 (11 : 130)	139.25 ± 19.33 (117 : 170)	125.5 ± 35.32 (64 : 171)
11	21.5 ± 38.66 (−38 : 60)	74.38 ± 42.41 (31 : 134)	40.88 ± 30.81 (10 : 108)
12	19.75 ± 57.47 (−62 : 80)	70.88 ± 27.35 (45 : 115)	60.5 ± 30.87 (33 : 127)
13	−24.63 ± 46.73 (−94 : 24)	49.75 ± 23.31 (24 : 87)	26 ± 37.24 (-33 : 66)
14	−10.63 ± 44.94 (−79 : 34)	49.13 ± 25.65 (25 : 89)	63.88 ± 28.81 (29 : 121)
15	90.13 ± 56.23 (17 : 149)	186.88 ± 53.36 (132 : 253)	154.38 ± 30.79 (129 : 222)
16	93 ± 48.62 (21 : 143)	167 ± 24.64 (140 : 206)	155.25 ± 32.34 (115 : 225)
17	195.75 ± 31.58 (133 : 228)	214.88 ± 12.87 (197 : 234)	178.5 ± 70.41 (102 : 300)
18	123.38 ± 42.81 (55 : 172)	191.25 ± 26.18 (164 : 228)	187.38 ± 31.33 (154 : 249)
Total	50.22 ± 70.27 (−94 : 228)	113.26 ± 59.84 (24 : 253)	91.83 ± 60.86 (−33 : 300)
Histogram	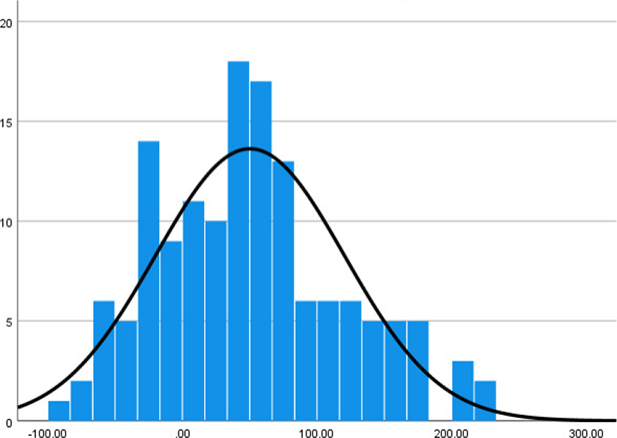	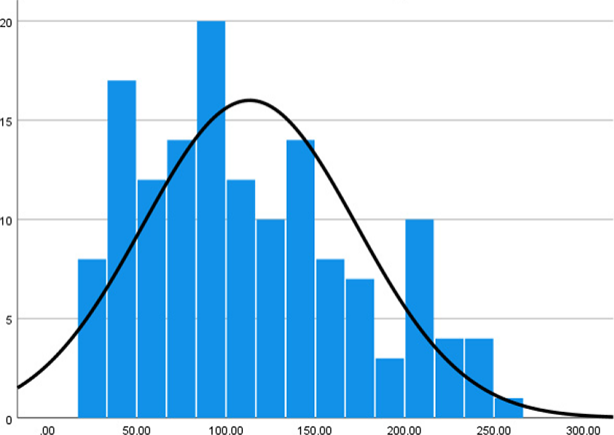	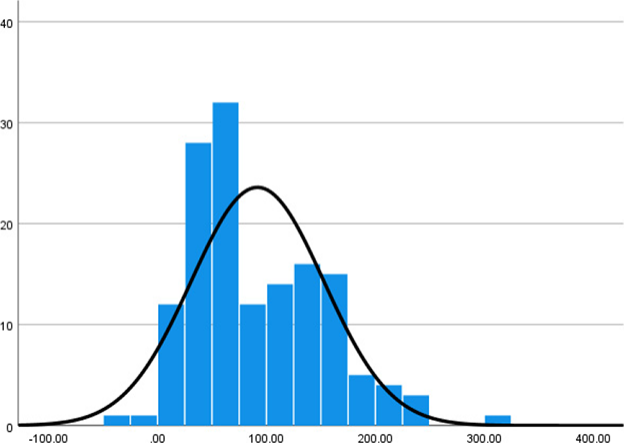

**Table 3:** Mean measurements of the metal substrcutrues stratified by zones (D–F).

	Zone D	Zone E	Zone F
ID	μ ± SD (min : max)	μ ± SD (min : max)	μ ± SD (min : max)
1	44.88 ± 32.61 (13 : 93)	27.63 ± 11.58 (14 : 43)	31.63 ± 43.32 (−10 : 108)
2	35 ± 18.74 (15 : 64)	21 ± 14.13 (2 : 39)	36 ± 42.84 (−1 : 111)
3	66 ± 34.16 (36 : 118)	47.5 ± 26.65 (14 : 79)	49.63 ± 48.21 (4 : 130)
4	59.5 ± 32.14 (31 : 109)	28.38 ± 21.99 (−4 : 54)	47.38 ± 39.82 (9 : 117)
5	26.88 ± 56.47 (−30 : 113)	8.13 ± 11.83 (−6 : 23)	32.5 ± 44.24 (−11 : 100)
6	36.88 ± 43.61 (−17 : 96)	17.75 ± 26.49 (−14 : 52)	34.75 ± 48.24 (−12 : 118)
7	140.63 ± 30.18 (110 : 193)	119 ± 28.71 (84 : 152)	115.75 ± 42.71 (74 : 182)
8	41.75 ± 35.99 (10 : 96)	35.13 ± 28.43 (1 : 70)	37.75 ± 39.47 (2 : 109)
9	49 ± 45.07 (10 : 121)	14.38 ± 21.01 (−12 : 40)	21.13 ± 28.71 (−5 : 73)
10	122.88 ± 32.04 (90 : 172)	100.25 ± 33.98 (54 : 136)	87.88 ± 21.79 (63 : 136)
11	42 ± 21.92 (6 : 76)	28.63 ± 17.63 (4 : 50)	27 ± 50.11 (−23 : 103)
12	16.88 ± 27.42 (−6 : 68)	18 ± 26.75 (−15 : 51)	36.5 ± 36.29 (2 : 98)
13	13.88 ± 22.31 (−13 : 51)	9.13 ± 15.27 (−12 : 30)	31.38 ± 38.64 (−4 : 103)
14	27.63 ± 34.87 (−2 : 86)	15.38 ± 25.69 (−16 : 46)	9.5 ± 41.47 (−30 : 79)
15	136 ± 35.12 (108 : 198)	90.5 ± 20.78 (58 : 113)	101.5 ± 35.98 (67 : 162)
16	90 ± 25.53 (68 : 136)	73.88 ± 24.86 (42 : 103)	62.75 ± 43.96 (25 : 140)
17	100.38 ± 28.04 (72 : 150)	87.63 ± 34.04 (51 : 125)	107 ± 51.62 (53 : 176)
18	140.63 ± 32.7 (106 : 195)	102.13 ± 29.51 (69 : 135)	113.38 ± 43.73 (62 : 178)
Total	66.15 ± 53.41 (−30 : 198)	46.91 ± 43.08 (−16 : 152)	54.63 ± 51.67 (−30 : 182)
Histogram	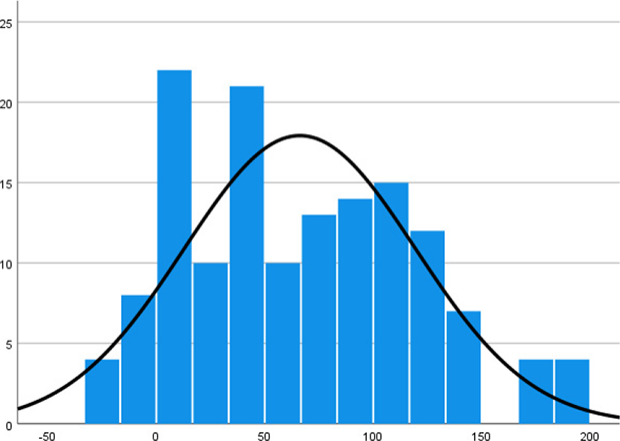	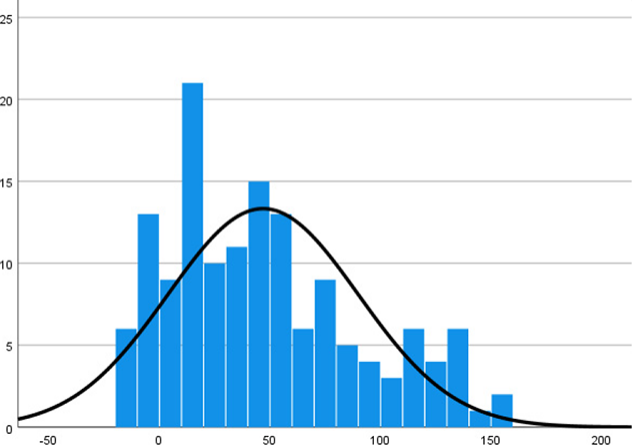	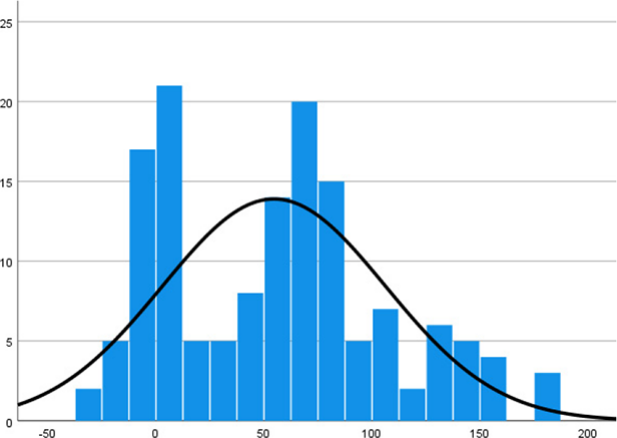

**Table 4:** Mean measurements of the metal substrcutrues stratified by zones (G–H).

	Zone G	Zone H
ID	μ ± SD (min : max)	μ ± SD (min : max)
1	50.63 ± 20.06 (28 : 86)	39 ± 43.49 (−28 : 105)
2	43.5 ± 20.11 (19 : 74)	8.13 ± 39.4 (−45 : 66)
3	62 ± 17.1 (42 : 89)	38.13 ± 40.01 (−14 : 110)
4	55.75 ± 25.63 (26 : 95)	20.25 ± 42.68 (−39 : 86)
5	38 ± 14.71 (20 : 58)	4.63 ± 40.88 (−46 : 64)
6	75.25 ± 25.79 (51 : 118)	57.25 ± 40.31 (7 : 116)
7	115.88 ± 15.42 (101 : 146)	93.88 ± 42.46 (33 : 161)
8	54 ± 25.5 (23 : 92)	4.5 ± 42.25 (−49 : 64)
9	41 ± 12.68 (31 : 68)	4.88 ± 45.76 (−63 : 80)
10	86.63 ± 39.34 (40 : 147)	66.25 ± 39.52 (18 : 132)
11	64.5 ± 21.13 (41 : 101)	39.38 ± 43.5 (−16 : 97)
12	61.75 ± 26.63 (31 : 120)	20.13 ± 42.36 (−33 : 79)
13	37.63 ± 17.94 (14 : 67)	−11.38 ± 40.85 (−63 : 51)
14	35.38 ± 19.39 (10 : 70)	17.38 ± 39.97 (−39 : 72)
15	92 ± 34.62 (53 : 135)	45.13 ± 47.69 (−33 : 122)
16	104 ± 27.08 (63 : 146)	87.25 ± 43.27 (27 : 151)
17	169.88 ± 23.62 (133 : 218)	182.63 ± 54.95 (105 : 278)
18	128.13 ± 21.44 (105 : 160)	122.88 ± 37.59 (75 : 189)
Total	70.31 ± 42.19 (10 : 218)	46.68 ± 62.61 (−63 : 278)
Histogram	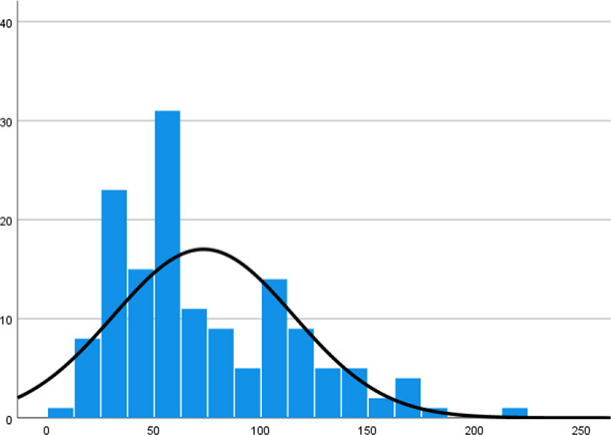	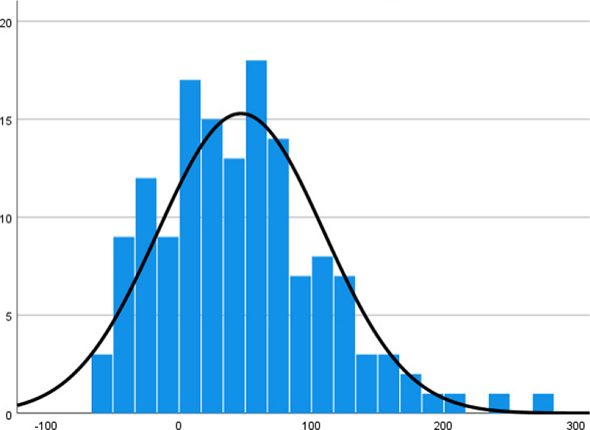

Some zones showed better results than others; regarding Forward VMD the worst zone is A, whereas regarding Backward VMD, the worst zone is B (Table 5). This is matching with Table 6 where the worst zone regarding the level of Forward VMD is zone A, while regarding the level of Backward VMD beyond 100 μm is zone B. Spearman’s correlation test shows the correlation between the level of Forward VMD and the level of Backward VMD beyond 100 μm in all the zones (Table 7). Moreover, Spearman’s correlation test shows the correlation between the zones in terms of the level of VMD (Table 8). Table 9 shows mean of the level of VMD in the substructures milled on each day of the week.

**Table 5:** Frequencies and mean measurements of zones discrepancies (A–H).

Zone	Forward VMD		Backward VMD beyond 100 μm	
	*N* (%)	μ ± SD (min : max)	*N* (%)	μ ± SD (min : max)
A	36 (25)	−35.28 ± 22.81 (−94 : −2)	32 (22.2)	152.84 ± 34.46 (102 : 228)
B	0 (0)		76 (52.7)	158.93 ± 44 (100 : 253)
C	2 (1.4)	−23.5 ± 13.44 (−33 : −14)	58 (40.3)	154.34 ± 41.28 (101 : 300)
D	12 (8.3)	−11.33 ± 10.6 (−30 : −1)	42 (29.2)	132.64 ± 28.18 (102 : 198)
E	19 (13.2)	−7.79 ± 4.76 (−16 : −1)	22 (15.3)	124.23 ± 15.181 (100 : 152)
F	24 (16.7)	−10.96 ± 8.85 (−30 : −1)	27 (18.8)	134.93 ± 23.68 (100 : 182)
G	0 (0)		41 (28.5)	129.95 ± 26.58 (101 : 218)
H	33 (22.9)	−29.3 ± 16.94 (−63 : −3)	27 (18.8)	143.74 ± 42.66 (105 : 278)
Total	126 (10.9)	−22.47 ± 19.44 (−94 : −1)	325 (28.2)	144.85 ± 37.53 (100 : 300)

**Table 6:** Level of Backward VMD beyond 100 μm, level of Forward VMD and level of VMD.

Zone	Level of Forward VMD	Level of Backward VMD beyond 100 μm	Level of VMD
A	36	32	68
B	0	76	76
C	2	58	60
D	12	41	53
E	19	22	41
F	24	27	51
G	0	41	41
H	33	27	60

**Table 7:** Correlation between the level of Forward VMD and the level of Backward VMD beyond 100 μm in all the zones. The Spearman correlation (ρ) test was used with a significance level of <0.05; 0.

			Level of Forward VMD					
			A	C	D	E	F	H
Level of Backward VMD beyond 100 μm
	A	ρ.	−0.788	−0.187	−0.471	−0.607	−0.659	−0.742
		Sig.	<0.001	0.457	0.048	0.008	0.003	<0.001
	B	ρ.	−0.867	−0.388	−0.576	−0.641	−0.621	−0.780
		Sig.	<0.001	0.112	0.012	0.004	0.006	<0.001
	C	ρ.	−0.828	−0.405	−0.501	−0.558	−0.540	−0.866
		Sig.	<0.001	0.096	0.034	0.016	0.021	<0.001
	D	ρ.	−0.675	−0.247	−0.484	−0.415	−0.613	−0.559
		Sig.	0.002	0.324	0.042	0.087	0.007	0.016
	E	ρ.	−0.779	−0.168	−0.422	−0.543	−0.589	−0.746
		Sig.	<0.001	0.506	0.081	0.020	0.010	<0.001
	F	ρ.	−0.707	−0.027	−0.425	−0.638	−0.443	−0.605
		Sig.	0.001	0.916	0.079	0.004	0.066	0.008
	G	ρ.	−0.750	−0.226	−0.247	−0.425	−0.456	−0.829
		Sig.	0.000	0.368	0.324	0.079	0.057	<0.001
	H	ρ.	−0.900	−0.228	−0.404	−0.564	−0.472	−0.883
	Sig.	<0.001	0.364	0.096	0.015	0.048	<0.001

**Table 8:** Correlation between the zones in term of level of VMD.

		A	B	C	D	E	F	G	H
A	ρ.	1.000	0.314	0.337	0.450	0.397	−0.208	0.316	0.688**
	Sig.		0.204	0.172	0.061	0.103	0.408	0.201	0.002
B	ρ.	0.314	1.000	0.621**	0.584*	0.321	−0.031	0.710**	0.019
	Sig.	0.204		0.006	0.011	0.194	0.903	0.001	0.941
C	ρ.	0.337	0.621**	1.000	0.560*	0.605**	0.044	0.861**	0.212
	Sig.	0.172	0.006		0.016	0.008	0.861	0.000	0.399
D	ρ.	0.450	0.584*	0.560*	1.000	0.821**	0.083	0.644**	0.233
	Sig.	0.061	0.011	0.016		0.000	0.743	0.004	0.352
E	ρ.	0.397	0.321	0.605**	0.821**	1.000	0.207	0.656**	0.313
	Sig.	0.103	0.194	0.008	0.000		0.410	0.003	0.206
F	ρ.	−0.208	−0.031	0.044	0.083	0.207	1.000	0.180	−0.015
	Sig.	0.408	0.903	0.861	0.743	0.410		0.475	0.952
G	ρ.	0.316	0.710**	0.861**	0.644**	0.656**	0.180	1.000	0.184
	Sig.	0.201	0.001	0.000	0.004	0.003	0.475		0.466
H	ρ.	0.688**	0.019	0.212	0.233	0.313	−0.015	0.184	1.000
	Sig.	0.002	0.941	0.399	0.352	0.206	0.952	0.466	

**Notes:**

* Correlation is significant at the 0.05 level (2-tailed).

** Correlation is significant at the 0.01 level (2-tailed).

**Table 9:** The total level of VMD and mean of the substructures milled on each day of the week.

Day	Total level of VMD	*n*	Mean
Mondays	35	5	7
Tuesdays	21	3	7
Wednesdays	27	4	6.75
Thursdays	25	4	6.25
Fridays	12	2	6
Total	35	18	7

## Discussion

### Reproducibility of CAD/CAM restorations

Although all of the received substructures were fabricated from the same scan, they were not identical and varied widely among each other. This means that the reproducibility of CAD/CAM restorations is questionable. The differences among the results of the 18 restorations arose from the laboratory procedures such as calibration of the milling machines and wearing of the cutting parts, whereas the differences among the results of the eight zones might be related not only to the laboratory procedures but also to the anatomy of the zone and the preparation of the finish line at that zone. These differences can be markedly noticed in terms of mean measurements in μm and the level of VMD.

### Marginal fit

Results demonstrated that Backward VMD was less than 100 μm, these results agree with a systematic review that included 90 studies and found that it is possible to obtain a tooth–prosthesis gap less than 80 μm by using CAD/CAM technology (Boitelle et al., 2014) bearing in mind that Backward VMD is always equal to or greater than the marginal gap. Therefore, the substructures are considered within the clinically acceptable range in terms of Backward VMD. However, the clinical goal is still not achieved and adjustments on the master cast might decrease the Backward VMD. The clinical goal ranges between 25 and 40 µm for cemented restorations (Sakrana, 2013; Papadiochou & Pissiotis, 2018). Backward VMD beyond 100 μm was detected in at least one measurement in 88 zones out of 144 zones. The extent of the improvement can only be measured after the adjustment since Backward VMD is a result of several factors, some of them are correctable while others are not. Forward VMD, which can be corrected to a certain limit in all cases by using diamond burrs, was detected in at least one measurement in 44 zones out of 144 zones. Thus, adjustments on the master cast can result in a 30.5% (44/144) improvement of the marginal fit in terms of Forward VMD.

The adjacent zones A and H, which correspond with buccal and disto-buccal zones, had the greatest forward VMD. On the other hand, the adjacent zones B and C, which correspond with buccal and mesio-buccal zones, had the highest backward VMD. Further investigations are needed to explain why the buccal zones are worse than the other zones.

Although a strong correlation may exist between the level of Backward VMD beyond 100 μm and the level of Forward VMD in the same zone like in zone H (ρ = −0.883), it cannot be taken as a rule that the level of Backward VMD can positively or negatively influence the level of Forward VMD in the same zone or even in the adjacent zone since the correlations differ substantially. Similarly, the correlations between the VMD in different zones varies between ρ −0.208 and ρ 0.861, and it cannot be taken as a rule that the extent of the VMD in one zone can positively or negatively influence the level of VMD in the same zone or even in the adjacent zone.

### The effect of the day of the week on the marginal fit

No clear evidence can be synthesized from this study regarding the correlation between the level of VMD of the substructure manufactured by CAD/CAM and the day of the week in which milling occurred due to proximity of the level of VMD in different days and due to the limited number of samples.

### Conjecture

The indirect digitalization with the master cast has the advantage of adjusting the margins of the CAD/CAM restorations extra-orally and consequently achieving a better marginal fit. On the other hand, direct digitalization has advantages in reducing impression time and patient comfort (Takeuchi et al., 2018).

Further research is necessary to investigate the improvement in reducing the marginal gap after adjustments on the master cast and whether achieving Backward VMD ranging 25 to 40 μm is possible. Further investigations are also required to relate the quality of the substructure and the day of the week since the limitation of this study in terms of sample size didn’t reveal an answer.

## Conclusions

Although all the restorations were fabricated from the same scan, they were not identical and varied widely among each other. Within the limitations of this *in vitro* study, it can be concluded that the marginal fit of CAD/CAM metal restorations is clinically acceptable. However, it still can be improved by the extraoral adjustments of unavoidable marginal discrepancy on the master cast. Thus, dispensing with the master cast deprives the dentist of delivering a restoration with higher quality. No clear evidence can be synthesized from this study regarding the correlation between the level of VMD of the substructure manufactured by CAD/CAM and the day of the week in which the milling process was made. Feedback from the dental office to the laboratory would help in detecting the causes of the discrepancies and improving the quality of the restorations manufactured.

## Supplemental Information

10.7717/peerj.13280/supp-1 Supplemental Information 1 Raw data of 1,152 measures. Measurements of all 18 metal substructure.
Metal substructures are given numbers from 1 to 18.
Zones are given letters from A to H.
Two photomicrographes were taken for each zone.
Each photomicrograph was measured at 4 reference points.

## Additional Information and Declarations

### Data Availability

The following information was supplied regarding data availability: The raw measurements are available in the Supplemental File.
